# Associations between volatile fatty acid profiles, methane emissions, and rumen microbiota in sheep fed Ethiopian forage

**DOI:** 10.3389/fmicb.2025.1731623

**Published:** 2026-01-22

**Authors:** Wondimagegne Bekele, Lovely Mahawar, Mohammad Ramin, Addis Simachew, Benedicte Riber Albrectsen, Abiy Zegeye

**Affiliations:** 1 Department of Applied Animal Science and Welfare, Swedish University of Agricultural Sciences, Umeå, Sweden; 2 Biotechnology Research Center, Institute of Advanced Science and Technology, Addis Ababa University, Addis Ababa, Ethiopia; 3 Department of Animal Science, Debre Berhan University, Debre Birhan, Ethiopia; 4 Department of Plant Physiology, Umeå Plant Science Centre, Umeå University, Umeå, Sweden

**Keywords:** Archaea, Bacteria, CH_4_ intensity, gut microbiota, Illumina sequencing, metabarcoding, ruminant, volatile fatty acids

## Abstract

This study was part of an *in vivo* investigation of methane (CH_4_) abatement feed on local Menz breed sheep in Ethiopia, conducted over 90 days period using a randomized complete block design. Sheep were subjected to four dietary treatments: Control, Acacia (*Acacia nilotica*), BSG (Brewer's Spent Grain), and Ziziphus (*Ziziphus spina-christi*). The aim of the study was to investigate the rumen microbial community composition, diversity, and their relationships with CH_4_ intensity. Rumen fluid was collected on days 0 (SD_0), 45 (SD_45), and 90 (SD_90), using an esophageal tube. The dynamics of the bacterial and archaeal domains were assessed by 16S rRNA gene sequencing. The sequencing results showed that 92.9% of ASVs were Bacteria, and 0.05% Archaea. At the genus level, *Rikenellaceae RC9 gut* group (18%), *Prevotella* (17%), and *Candidatus* Saccharimonas (8.9%) were the most abundant Bacteria, while *Methanobrevibacter* (88%) dominated the Archaeal genera across all treatment groups. Treatment feed significantly altered microbial profiles, notably reducing *Methanobrevibacter* abundance in CH_4_ abatement diets and increasing the presence of *Methanosphaera*. Shannon diversity increased in the abatement diet and decreased when the sheep were fed BSG. CH_4_ intensity was most strongly associated with the archaeal genus *Methanomicrobium*, but did not associate strongly with any other Bacteria or Archaea, although *Methanobrevibacter* and *Methanosphaera* were correlated negatively (*r* = –0.97). CH_4_ intensity also did not covary with volatile fatty acids (VFAs), of which Acacia yielded the highest acetate (772 mmol/mol) and BSG the highest propionate (172 mmol/mol) concentration. The volatile fatty acids (VFAs) showed a strong correlation: a positive correlation between acetate and butyrate (*r* = 0.80) and a strong negative correlation between acetate and propionate (*r* = –0.92). These findings highlight the complex relationship between diet, rumen microbiota, and fermentation products, with implications for CH_4_ mitigation strategies in sheep.

## 1 Introduction

Ruminants are animals that digest plant-based food through a specialized four-compartment stomach, the rumen, reticulum, omasum, and abomasum (Balch, 1959; Wang et al., 2018; Horstmann, 2021). Rumen is one of the most underexplored microbial ecosystems that produce a diverse range of enzymes for digesting and utilizing various plant constituents (Goel et al., 2015). These enzymes are produced by a diverse community of Bacteria, Protozoa, Archaea, Fungi, and Bacteriophages residing in the rumen in a symbiotic relationship (Sahu et al., 2017; Gruninger et al., 2018). This diverse group of anaerobic microorganisms collaborates to decompose dietary components in the feed that the host animal cannot digest, producing volatile fatty acids that facilitate improved feed digestion (Hobson and Stewart, 1997). Furthermore, rumen microorganisms can influence nutrient absorption and are likely significant factors in nutrient utilization efficiency and detoxifying plant secondary compounds (John Wallace, 2008). The rumen microbiome is crucial for ruminant physiology, pathology, and host immunity (Li, 2015). However, they also contribute to CH_4_ production (Morgavi et al., 2013).

Methane is the second-largest greenhouse gas contributing to global warming, after CO_2_. It is 28 times more potent than CO_2_ over 100 years and 80 times more potent over 20 years (EPA (U.S. Environmental Protection Agency), 2020). Animal agriculture makes up a significant part of its emissions (Laubach et al., 2024). In Ethiopia, sheep constitute 26% of the national ruminant population and produce an estimated 7% of the country’s total enteric CH_4_ emissions (Kariuki et al., 2024). To limit global warming by 2030, countries should cut CH_4_ emissions, especially from livestock, by 30% (FAO (Food and Agriculture Organization of the United Nations), n.d.). Estimates suggest that over 87% of the CH_4_ generated by sheep originates from the rumen (Murray et al., 1976). Thus, the ruminal microbiome probably plays a role in determining the host’s CH_4_ production phenotype (Shi et al., 2014). Understanding the differences in the structure of rumen microbial communities is essential for identifying the factors that lead to low CH_4_ production (Kittelmann et al., 2014). By examining the role of rumen microbiota and the connections between microbiomes and animal traits, we can choose livestock farming practices that minimize greenhouse gas emissions (Tapio et al., 2017).

Traditional microbial culture-dependent techniques have been superseded by faster and potentially more accurate molecular methods (de Silva Oliveira et al., 2007). The rise of culture-independent approaches, such as metabarcoding, offers a valuable way to investigate the impact of rumen microbial communities on rumen biological processes (Gruninger et al., 2018; Bekele et al., 2021). DNA metabarcoding utilizes sequence analysis of conserved gene regions, enabling fast and reliable identification of species diversity in complex biological communities (Abdi et al., 2024). Many phylogenetic tools used for microbial classification rely on the ribosomal 16S rRNA gene, a marker gene for determining prokaryotic community composition and diversity (de Silva Oliveira et al., 2007). The 16S rRNA gene sequences are widely used because they are present in nearly all prokaryotes, exist as a multi-gene family or operons, and have a conserved function over time. Additionally, the 16S rRNA gene (about 1,500 bp) is sufficiently large for bioinformatics applications (Patel, 2001; Janda and Abbott, 2007).

This study investigates the composition and diversity of rumen Bacteria and Archaea and their relation to CH_4_ intensity and VFA production. We hypothesized that microbial profiles would vary based on dietary treatment, assisting in identifying specific rumen microbes that significantly influence CH_4_ intensity in Menze sheep. Menz sheep breed is one of Ethiopia’s 14 sheep types, well-adapted to the cold highland climate. It tolerates drought and seasonal feed fluctuations, resists endo-parasite infections, and produces meat, coarse wool, skin, and manure (Tibbo et al., 2004; Getachew, 2008).

The feeds used in the *in vivo* study were obtained from previous research on screening ruminant feedstuffs for low *in vitro* CH_4_ yield and optimal nutrient composition. Among the top feeds with low CH_4_ yields were *Acacia nilotica* (L.) (6.6 g of CH_4_/kg DM), Ziziphus spina-christi (7.8 g of CH_4_/kg DM), and Brewer’s spent grain (8.1 g of CH_4_/kg DM). Details of the characterization of the feed resources can be found in Bekele et al. (2024).

## 2 Materials and methods

### 2.1 Animal handling, diet, and sampling

This study was part of an *in vivo* evaluation of a promising CH_4_ abatement feed in the diet of local sheep. Bekele et al. (2025) provides a detailed description of the *in vivo* study. Briefly, 21 yearling Menz sheep with a mean initial live body weight of 22.7 ± 1.7 kg (mean ± SD) were used. The candidate feed for the *in vivo* study consisted of dried leaves of *Acacia nilotica*, *Ziziphus spina-christi*, and BSG (Table 1). The experiment was designed as a randomized complete block design and spanned 90 days.

**Table 1:** Proportion of treatment feed ingredients on a dry matter (DM) basis for the *in vivo* trial.

Treatments	Wheat bran (g)	Noug seed cake (g)	Test feed (g)	Grass hay (g)	CH_4_ intensity
Control	200	200	0	*Ad libitum*	825
Acacia	100	100	304	*Ad libitum*	391
BSG	100	100	243	*Ad libitum*	478
Ziziphus	100	100	380	*Ad libitum*	265
CH4 intensity (g CH_4_/kg ADG (average daily gain))					

Source: Bekele et al. (2025).

Rumen fluid samples were collected from the experimental animals on sample days 0 (SD_0), 45 (SD_45), and 90 (SD_90). SD_0 represents samples collected before the treatment feed was introduced, while SD_45 and SD_90 correspond to samples collected on days 45 and 90 of the *in vivo* experiment following treatment initiation. The rumen fluids were collected in a Falcon tube using an oral stomach tube before the morning feed. The first ~30 mL of collected rumen fluids was discarded to minimize saliva contamination. A 50 mL sample was then collected and sieved through four layers of cheesecloth, then frozen in liquid nitrogen and stored at −20 °C for further analysis.

### 2.2 Volatile fatty acids

For VFA analysis, three replicates of 2.5 mL rumen fluid, along with 0.5 mL of 25% metaphosphoric acid, were prepared in 15 mL Falcon tubes for each sample. Then, the samples were pooled for each treatment to facilitate VFA analysis. VFAs in the rumen contents from each pooled sample were analyzed in the Department of Applied Animal Science and Welfare, Swedish University of Agricultural Sciences, Uppsala, Sweden, using HPLC described previously by Angellotti et al. (2025).

### 2.3 DNA extraction, PCR amplification, and sequencing method

eDNA from the rumen samples were extracted using the QIAGEN DNA kit (DNeasy PowerSoil Kit, USA). The region (V3–V4) of the 16S rRNA gene was amplified with the primer sequences 338\u00B0F (5′-ACTCCTACGGGAGGCAGCA-3′) and 806R (5′-GGACTACHVGGGTWTCTAAT-3′), which were overhang with the index sequences (5′-ACACTCTTTCCCTACACGACGCTCTTCCGATCT-338F and 5′-GTGACTGGAGTTCAGACGTGTGCTCTTCCGATCT-806R) to allow for dual indexing of the samples (Li et al., 2022). The extracted DNA was quantified with Qubit 2.0 Fluorometer (Life Technologies Invitrogen, USA). A total of 25 μL PCR mixture, consisting of 20 μL master mix with primer and BSA, 12.5 μL of KAPA HiFi HotStart ReadyMix, KK2601 (Roche sequencing store), 10 μM of each primer, 0.02 μg/μL of bovine serum albumin (BSA), and 5 μL of eDNA with a concentration of 2.5 ng/μL, was used for the PCR reaction programmed as follows: Initial activation at 95 °C for 3 min; denaturation at 95 °C for 30 s; annealing at 55 °C for 30 s; elongation at 72 °C for 30 s; and final elongation at 72 °C for 5 min, repeated for 25 cycles. The quality of PCR products was evaluated using 1.2% agarose gel electrophoresis.

### 2.4 Library preparation and amplicon sequencing

PCR 2 (indexing primer), 6 μL of DNA template (5 ng/μL); 10 μL of KAPA HiFi HotStart ReadyMix, KK2601, Roche; 4 μL of index primer mix (provided by NGI); and water as needed to reach a total volume of 20 μL were used. The PCR 2 cycling conditions were as follows: Initial activation at 98 °C for 2 min; denaturation at 98 °C for 20 s; annealing at 55 °C for 20 s; elongation at 72 °C for 15 s; final elongation at 72 °C for 2 min for 8 cycles. PCR products were checked on a 1.2% agarose gel. After confirming the successful attachment of sample-specific indexing primers, the amplicons were sent to Science for Life Laboratory (NGI SciLifeLab), Stockholm, Sweden, in cold conditions for library preparation and Illumina NextSeq2000 (300 bp) paired-end (PE) sequencing.

### 2.5 Bioinformatics analysis

The sequence quality was assessed using FastQC (Andrews, 2010), and the results were summarized with the FastQC module of MultiQC (Ewels et al., 2016) within the nf-core/ampliseq workflow (version 2.10.0). Primers were trimmed using Cutadapt (Martin, 2011) with cutadapt_min_overlap_3, and untrimmed sequences were discarded. Sequences lacking primer regions were considered artifacts. Adapter- and primer-free sequences were processed independently for each sample using DADA2 (Callahan et al., 2016) to remove low-quality reads (−-trunc_qmin 25) and PCR chimeras, thereby enhancing sequence recovery. The resulting amplicon sequencing variants (ASVs) were filtered using the Barrnap tool (Seemann, 2013) to retain only bacterial and archaeal ASVs by excluding those with taxonomic annotations containing “mitochondria” or “chloroplast.” Taxonomic classification of bacterial and archaeal ASVs was performed using DADA2 and QIIME2 (Bolyen et al., 2019), with the Silva 138.1 reference database (Quast et al., 2012).

Statistical analyses were performed using R version 4.4.1 (R Core Team, 2024). The alpha and beta diversities of the microbial communities were assessed using the phyloseq and vegan packages. Nonparametric Kruskal-Wallis tests were employed to assess differences in Shannon diversity influenced by treatment and time, with effect sizes reported as η^2^ (eta-squared). Additionally, beta diversity was assessed using principal coordinates analysis (PCoA) with the Bray–Curtis dissimilarity metric. A Permutational Multivariate Analysis of Variance (PERMANOVA) was performed with the adonis2 function from the vegan package to evaluate the effects of treatment and time on microbial community composition. Rarefaction curves were plotted using the rarecurve function. The significance level was set at *p* < 0.05. Microbial profile data were visualized using the ggplot2 package. The correlation between microbiome taxa, CH_4_ intensity, and VFAs was analyzed using a Spearman Correlation Matrix with the Hmisc package.

## 3 Results

### 3.1 Sequencing output

Barrnap classified 70,166 (92.9%) ASVs as Bacteria, 38 (0.05%) ASVs belonging to Archaea. The eDNA’s taxonomic classification suggested 27 bacterial phyla (with 866 genera) and 5 archaeal phyla (with 3 genera).

**Figure 1:**
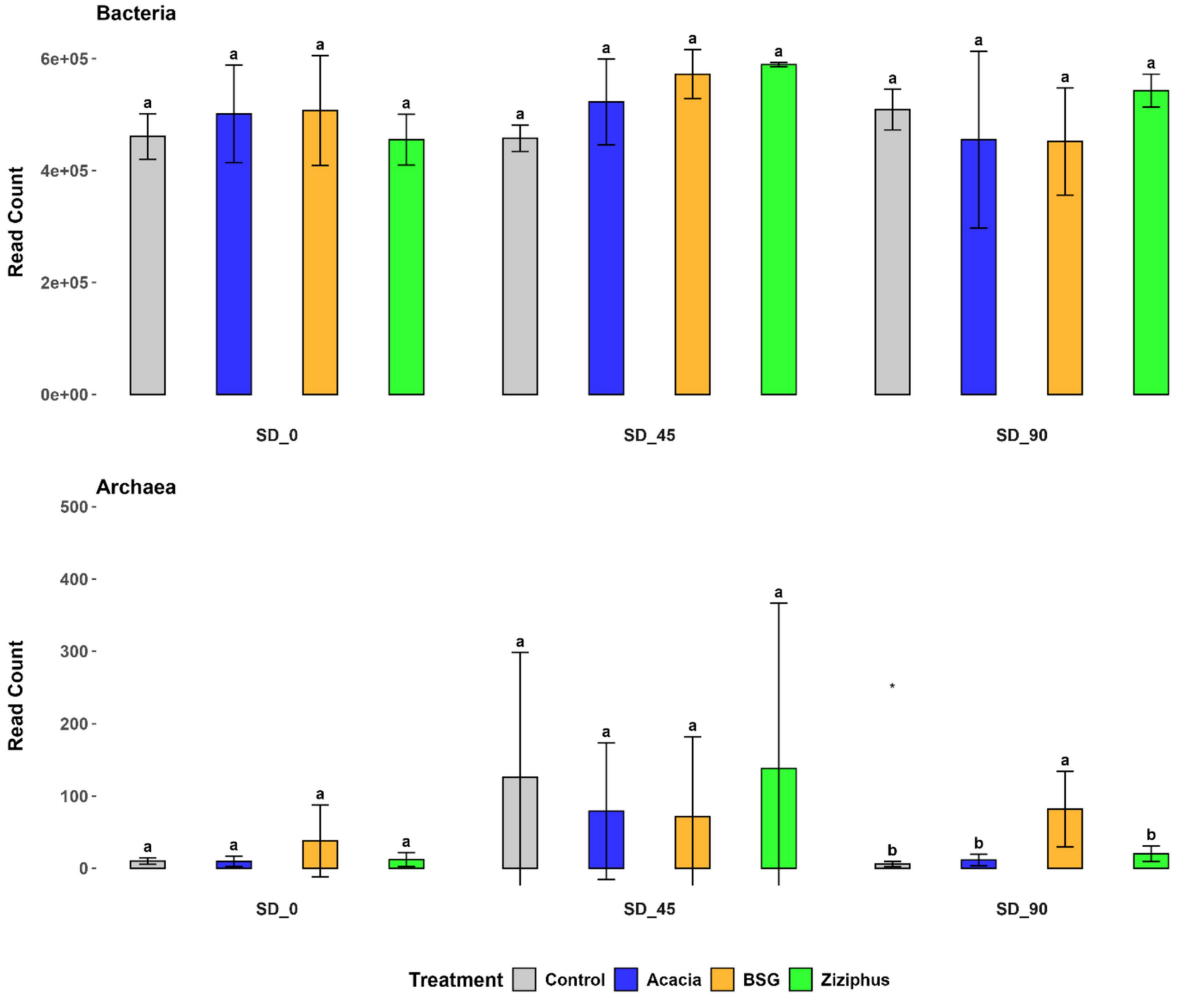
Read counts of Bacteria and Archaea from sheep rumen fluid eDNA after feeding four different diets (Control, Acacia, BSG, and *Ziziphus*) at three time points (SD_0, SD_45, and SD_90). SD_0 indicates samples collected before the treatment feed was introduced, while SD_45 and SD_90 represent samples taken on days 45 and 90 of the *in vivo* experiment after treatment started. eDNA was analyzed by amplifying the V3-V4 region of the 16S rRNA gene using primers 338F-806R, followed by sequencing on the Illumina NextSeq 2000. Differences in superscript letters indicate a significant difference at *p* < 0.05.

Rarefaction analysis revealed distinct ASV bacterial diversity patterns across treatments and time points. Amplicon sequence variant (ASV) bacterial diversity increased over time in all treatments except BSG. Both treatment and time affected microbial diversity, with Ziziphus showing the greatest increase (Supplementary Figure S1). The rarefaction curve was constructed only for Bacteria, while it was not possible to generate a rarefaction curve for archaeal diversity due to the small number of reads or library size. The read count did not show a significant difference across treatment and time points for Bacteria, but Acacia showed significantly (*p* < 0.05) higher read count at SD_90 for Archaea, as shown in Figure 1.

### 3.2 Rumen microbial community composition

**Figure 2:**
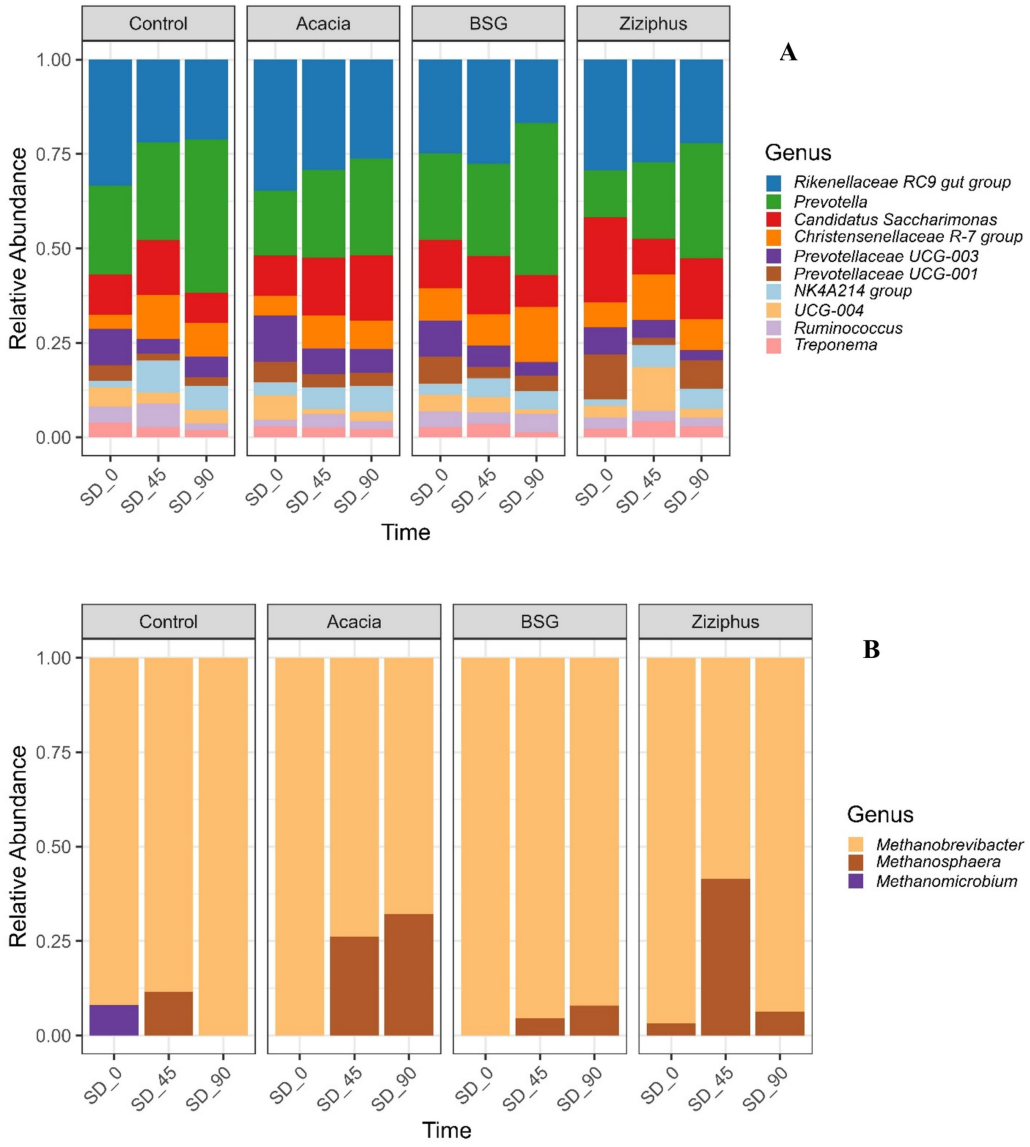
Genus-level composition of the rumen microbiota in sheep for each of the four diets (control, *Acacia*, BSG, and *Ziziphus*) across three time points (SD_0, SD_45, and SD_90) following the start of a feeding trial. SD_0 represents samples collected before the treatment feed was introduced, while SD_45 and SD_90 correspond to samples collected on days 45 and 90 following the *in vivo* treatment. **(A)** Relative abundance of bacterial genera. **(B)** Relative abundance of archaeal genera. For each time point, the bacterial and archaeal content (measured as ASVs of eDNA in the rumen juice sample) is normalized to 100% for each respective group.

Among the 27 bacterial phyla, the three most prevalent across different treatments and time points (Supplementary Figure S2) were *Bacteroidota*, *Firmicutes*, and *PatesciBacteria*, while among the 5 Archaea phyla, the dominant were *Euryarchaeota* (Supplementary Figure S3). Also, out of 866 bacterial genera, the three most abundant (Figure 2A) were *Rikenellaceae RC9 gut* group, *Prevotella*, and *Candidatus* Saccharimonas, while the archaeal genera included *Methanobrevibacter*, *Methanosphaera*, and *Methanomicrobium* (Figure 2B). Table S1, which displays the relative abundance of the top bacterial and archaeal phyla and genera across all samples, can be accessed in the Supplementary material section.

### 3.3 Rumen microbial community composition dynamics

Before administering the treatment feeds (SD_0), bacterial genera exhibited differences in their abundance. For example, the *Rikenellaceae RC9 gut* group abundance ranged from 16.6% (BSG) to 24.6% (Acacia), whereas *Prevotella* varied from 9.0% (Ziziphus) to 15.8% (Control). Furthermore, *Candidatus* Saccharimonas ranged from 7.2% (Control) to 16.2% (Ziziphus). Other genera, like the *Christensenellaceae R-7* group (2.6% in Control vs. 5.7% in BSG) and *Prevotellaceae UCG-001* (2.8% in Control vs. 8.6% in Ziziphus). Correspondingly, Archaea analysis for SD_0 yielded similar results as Bacteria. M*ethanobrevibacter* abundance ranged from 92% (Control) to 100% (in Acacia and BSG), while *Methanomicrobium* was only found in the Control at 8%. *Methanosphaera* was present solely in Ziziphus at 3.2%.

After administering the treatment feeds (SD_45 and SD_90), bacterial genera such as *Prevotella* increased across all treatments from SD_45 (14–17.2%) to SD_90 (17–27.4%), becoming the dominant genus in Control (27.1%) and BSG (27.4%) by SD_90, with steady increases in Acacia (15.7 to 17%) and Ziziphus (14 to 17.6%). *Rikenellaceae RC9 gut* group decreased across all treatments from SD_45 (14.6–19.7%) to SD_90 (11.4–17.4%), losing dominance relative to SD_0 (16.6–24.6%), particularly in BSG (11.4%) and Ziziphus (12.7%). Correspondingly, Acacia and Ziziphus induced significant shifts in archaeal composition. Acacia consistently reduced *Methanobrevibacter* (100 to 67.9%) and promoted sustained *Methanosphaera* growth (0 to 32.1%) over SD_45 to SD_90. Ziziphus caused a transient *Methanosphaera* peak at SD_45 (41.5%), followed by a sharp decline in *Methanobrevibacter* (58.5%), with subsequent recovery of *Methanobrevibacter* (93.8%) and a reduction in *Methanosphaera* (6.3%) by SD_90. Control showed transient *Methanosphaera* at SD_45 (11.6%) but reverted to complete *Methanobrevibacter* dominance (100%) by SD_90. BSG maintained high *Methanobrevibacter* levels (95.4 to 92.1%) with modest increases in *Methanosphaera* (4.6 to 7.9%).

### 3.4 Rumen microbial community diversity

**Figure 3:**
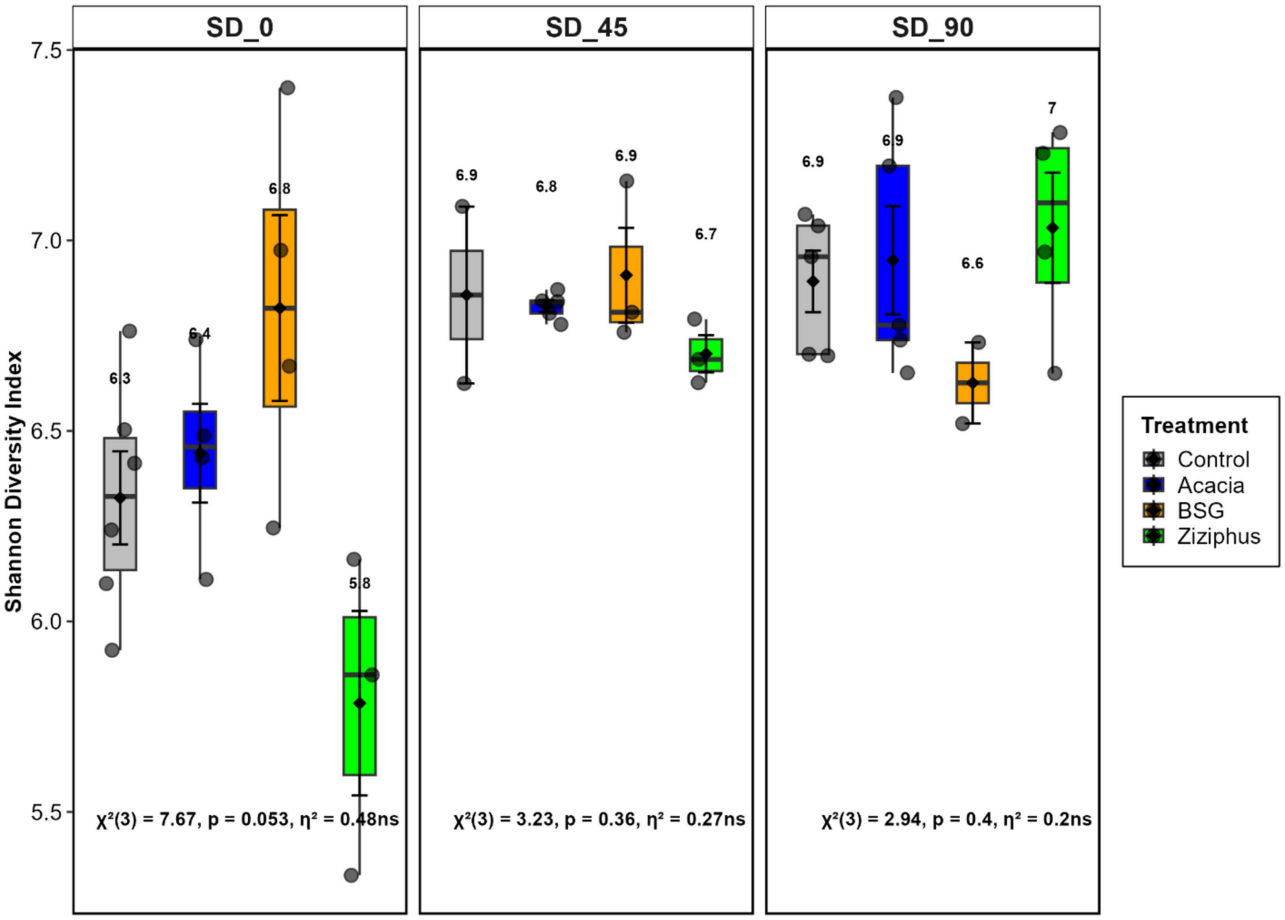
Alpha diversity of the microbial community eDNA from sheep rumen fluid after feeding of four diets (Control, Acacia, BSG, and *Ziziphus*) across three time points (SD_0, SD_45, and SD_90). SD_0 represents samples collected before the treatment feed was introduced, while SD_45 and SD_90 correspond to samples collected on days 45 and 90 of the *in vivo* experiment following treatment initiation.

**Figure 4:**
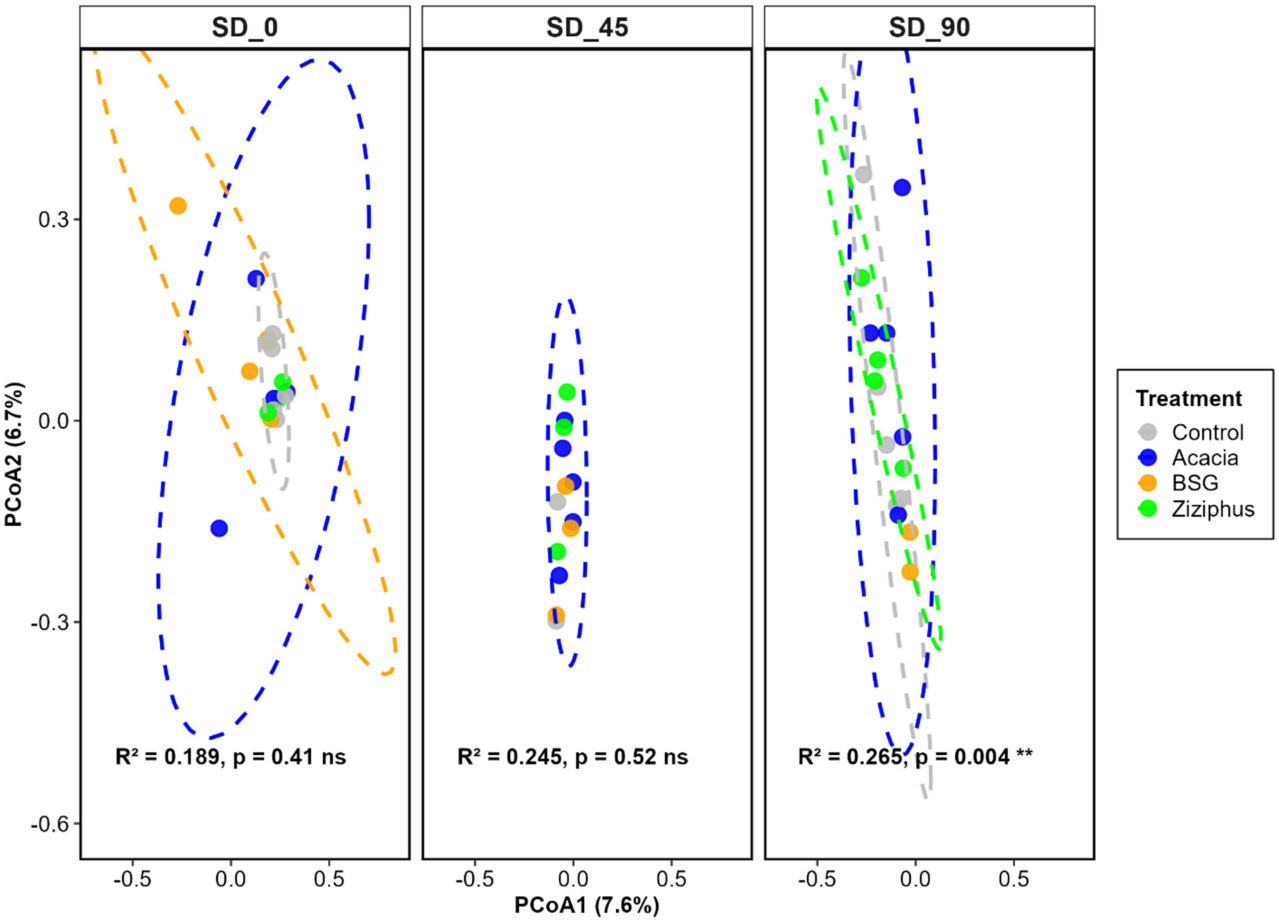
Beta diversity of microbial communities from eDNA of sheep rumen fluid after feeding of four diets (Control, Acacia, BSG, and *Ziziphus*) across three time points (SD_0, SD_45, and SD_90). SD_0 represents samples collected before the treatment feed was introduced, while SD_45 and SD_90 correspond to samples collected on days 45 and 90 of the *in vivo* experiment following treatment initiation. PCoA1 and PCoA2 represent the percentage of variation in community composition among samples, based on the Bray–Curtis distance matrix, that is captured by each axis.

The Shannon diversity index showed no significant differences among the four treatment groups at SD_0, SD_45, and SD_90, as shown in Figure 3. A non-significant result indicates no meaningful association between treatment and Shannon diversity, though with a strong effect size (η^2^ = 0.2 to 0.48). The microbial composition, as measured by beta diversity, showed no significant differences up to the SD_45 time point. A significant difference (*p* < 0.01) was observed at SD_90, as illustrated in Figure 4. The proportion of variance explained by treatment increased over time, from 18.9% at SD_0 to 24.5% at SD_45 and 26.5% at SD_90. This indicates a growing influence of dietary treatments on microbiome composition as the *in vivo* trial progressed.

### 3.5 Volatile fatty acids production

Table 2 displays the VFAs production of sheep fed the treatment diet. Acetate concentrations varied significantly across treatments (*p* < 0.05), with means ranging from 758 to 772 mmol/mol. Acacia exhibited the highest acetate production at 772 mmol/mol, while BSG had the lowest at 758 mmol/mol. Propionate levels showed a highly significant treatment effect (*p* < 0.01), with means ranging from 151 to 172 mmol/mol. BSG had the highest propionate concentration at 172 mmol/mol. Butyrate concentrations also differed significantly across treatments, with means ranging from 69.5 to 77.3 mmol/mol, where BSG had the lowest at 69.5 mmol/mol.

**Table 2:** Volatile fatty acids production from sheep rumen fed four treatment diets (Control, Acacia, BSG, and Ziziphus).

Variables	Treatments				SEM	*P*-value
	Control	Acacia	BSG	Ziziphus		
Acetate mmol/mol	767^ab^	772^a^	758^b^	769^ab^	1.13	0.04
Propionate mmol/mol	156^b^	151^b^	172^a^	158^b^	0.85	0.003
Butyrate mmol/mol	77.3^a^	76.9^a^	69.5^b^	73.8^a^	0.45	0.01

mmol/mol: millimoles per mole; SEM: standard error of mean; P = probability; ^ab^ = Means in the same row without common letter are different at *p* < 05. The post hoc method was Duncan’s Multiple Range Test (DMRT).

### 3.6 Relation between rumen microbes, CH_4_ intensity, and VFA

The correlation matrix in Tables 3, 4 illustrates the relationships among rumen bacterial and archaeal genera, as well as CH_4_ intensity and VFAs. There were weak associations and no significant correlations between CH_4_ intensity and bacterial genera. Additionally, associations between VFAs and bacterial genera were weak (Table 3). Among archaeal genera, *Methanosphaera* showed strong negative correlations with *Methanobrevibacter* (r = −0.973, *p* < 0.001) and a moderate negative correlation with *Methanomicrobium* −0.066. CH_4_ intensity positively correlated with *Methanomicrobium* (*r* = 0.483, *p* < 0.01). Conversely, CH_4_ intensity exhibited weak, non-significant correlations with *Methanobrevibacter* (*r* = 0.080), *Methanosphaera* (*r* = −0.195). Strong interrelationships were noted among VFAs. Acetate displayed a robust negative correlation with propionate (*r* = −0.970, *p* < 0.001) and a strong positive correlation with butyrate (*r* = 0.798, *p* < 0.001). Likewise, propionate and butyrate were strongly negatively correlated (*r* = −0.920, *p* < 0.001). Generally, the associations between VFAs and archaeal genera were weak and non-significant. Despite the robust interdependence of the VFAs, their connections to archaeal genera and CH_4_ intensity remained weak (Table 4).

**Table 3:** Spearman correlation matrix of rumen bacterial genera, CH_4_ intensity, and VFAs.

	1	2	3	4	5	6	7	8	9	10	11	12	13	14
(1) *Candidatus Saccharimonas*														
(2) *Christensenellaceae R-7 group*	0.16													
(3) *NK4A214 group*	0.06	**0.54** ^***^												
(4) *Prevotella*	**−0.55** ^***^	−0.14	0.14											
(5) *Prevotellaceae UCG-001*	0.26	−0.03	**−0.49** ^***^	**−0.38** ^*^										
(6) *Prevotellaceae UCG-003*	−0.03	−0.29	**−0.51** ^***^	**−0.32** ^*^	**0.33** ^*^									
(7) *Rikenellaceae RC9 gut group*	−0.05	**−0.51** ^***^	−0.28	**−0.35** ^*^	0.02	**0.36** ^*^								
(8) *Ruminococcus*	0.10	**0.30** ^*^	0.05	−0.25	0.13	0.08	−0.22							
(9) *Succiniclasticum*	0.01	**−0.30** ^*^	−0.42^**^	−0.10	0.10	**0.46** ^**^	0.25	0.12						
(10) *UCG-004*	−0.19	−0.18	−0.11	−0.16	−0.14	0.05	0.27	−0.28	−0.16					
(11) CH_4_ intensity	−0.18	−0.01	0.01	0.19	−0.18	0.08	−0.09	0.15	0.09	0.12				
(12) Acetate	0.16	−0.19	0.16	−0.22	−0.01	0.03	0.22	−0.23	0.09	0.03	−0.27			
(13) Propionate	−0.07	0.22	−0.16	0.13	0.08	−0.06	−0.23	0.20	−0.10	−0.02	0.10	**−0.97** ^***^		
(14) Butyrate	−0.07	−0.25	0.14	0.03	−0.18	0.10	0.21	−0.13	0.11	0.01	0.18	**0.80** ^***^	**−0.92** ^***^	

*p < 0.05, **p < 0.01, ***p < 0.001. Significant correlation values in bold.

**Table 4:** Spearman correlation matrix of rumen archaeal genera, CH_4_ intensity, and VFAs.

	1	2	3
(1) *Methanobrevibacter*			
(2) *Methanomicrobium*	−0.17		
(3) *Methanosphaera*	**−0.97** ^***^	−0.07	
(4) CH_4_ intensity	0.08	**0.48** ^**^	−0.20
(5) Acetate	−0.24	−0.03	0.25
(6) Propionate	0.20	−0.04	−0.20
(7) Butyrate	−0.11	0.13	0.08

Significant correlation values in bold. *p < 0.05, **p < 0.01, ***p < 0.001. For correlations among 4–7 (see Table 3).

## 4 Discussion

This study examined the rumen microbiome composition and diversity in local sheep fed CH_4_ abatement feed, as well as the link between microbiome abundance and rumen metabolites.

### 4.1 Rumen microbial community composition

Magne et al. (2020) and Cholewińska et al. (2021) reported that Phyla *Firmicutes* and *Bacteroidetes* are the most abundant in all ruminants and the human gut, which together represent more than 90% of the total microbial community. The *Firmicutes*/*Bacteroidetes* ratio influences health status and is considered a potential marker for metabolic diseases and feed inefficiency (dysbiosis). In a study by Langda et al. (2020), the diversity and composition of rumen microbes in goats and sheep in high-altitude environments were analyzed, identifying *Firmicutes* and *Bacteroidetes* as the predominant phyla, which aligns with our findings. The *Firmicutes* group is primarily responsible for breaking down exogenous peptides and amino acids. *Bacteroidetes* is characterized mainly by its ability to decompose cellulose, hemicellulose, and pectins in the ruminant digestive system (Cholewińska et al., 2021).

McLoughlin et al. (2023) also investigated the impact of breed (Cheviot (most efficient) and Connemara (least efficient)) and ruminal fraction on bacterial and archaeal community composition in sheep. They reported that *Prevotella* was the most common bacterial genus. Additionally, a study by Danielsson et al. (2017) examined the microbial composition of rumen archaeal and bacterial communities in dairy cows classified as high or low CH_4_ emitters. They identified *Euryarchaeota* and *Crenarchaeota* as the dominant phyla among all samples, with *Euryarchaeota* accounting for an average of 99.9 ± 7.2%. Among the Archaea sequences, the predominant genus was *Methanobrevibacter*, which represented 88.8%, followed by *Methanosphaera*. According to Janssen and Kirs (2008), most rumen Archaea (92.3%) are classified within the genera *Methanobrevibacter* and *Methanomicrobium*, consistent with our findings.

Out of 742 rumen samples, in the most comprehensive study, the dominant taxa were *Prevotella* (*Bacteroidetes*), *Butyrivibrio*, and *Ruminococcus* (all *Firmicutes*), as well as *Methanobrevibacter*, the core rumen microbe that thrives across all animal species and geographical regions sampled (Morgavi et al., 2010; Mizrahi and Jami, 2017).

### 4.2 Rumen microbial community composition dynamics

Before administering the treatment feeds (SD_0), the microbiome profiles were expected to be similar across groups since all sheep were subjected to identical pre-trial conditions. Despite this expectation, the data revealed significant differences, likely due to the individuality of the sheep. Variations in microbial populations can arise from these individual differences among sheep. Both genetic and physiological factors may contribute to the development of unique rumen microbiomes, influencing the baseline microbial composition even when environmental conditions remain stable (Rodríguez et al., 2015; Fernando et al., 2025). Mizrahi and Jami (2017) noted in their review that significant microbial differences persist among animals of similar lineages, even when raised under similar management conditions. Additionally, Welkie et al. (2010) observed that cows receiving identical diets can exhibit considerable disparities in their bacterial communities.

Sample processing may also limit the detection that contributes to variations in rumen microbes at SD_0. Denman and McSweeney (2019) noted differences in the relative abundances of certain Bacteria and Archaea using two sample processing methods.

After the administration of the treatment feeds (SD_45 and SD_90), Control, Acacia, BSG, and Ziziphus enhanced *Prevotella* growth (Control: +11.4%, Acacia: +4.8%, BSG: +12.2%, Ziziphus: +8.6% by SD_90) but reduced *Rikenellaceae RC9 gut* group (Control: −8.2%, Acacia: -7.2%, BSG: −5.1%, Ziziphus: −8.4% by SD_90), highlighting a consistent shift toward *Prevotella* dominance across all treatments. Betancur-Murillo et al. (2022) reported that *Prevotella* is central to carbohydrate and hydrogen metabolism, and a high abundance of *Prevotella* in ruminants is associated with a healthy microbiome. It is also capable of breaking down a variety of polysaccharides and can synthesize propionate, which in turn serves as the most important substrate for gluconeogenesis in the liver of ruminants. The consistent shift toward *Prevotella* may be due to the higher intake of neutral detergent fiber (NDF) from our *in vivo* study (569 to 633 g/day).

Acacia supported *Candidatus saccharimonas*, while Ziziphus caused dynamic shifts, including transient *UCG-004* and *Christensenellaceae R-7* group peaks, highlighting dietary responses. This variation was also observed in archaeal genera; the control group exhibited an increased abundance of *Methanobrevibacter*, whereas the other test feeds effectively suppressed it, specifically at SD_45. Bharathi et al. (2020) reported that *Methanobrevibacter* uses H_2_, CO_2_, and formate as substrates for CH_4_ production in ruminants. The enteric CH_4_ emitted by this microorganism can also significantly contribute to the loss of dietary energy in ruminants. The decrease in *Methanobrevibacter* in the test diet might result from the presence of a bioactive compound that specifically reduces substrates like H_2_ and CO_2_ used by *Methanobrevibacter*.

Ellison et al. (2017) reported that diet has a significant influence on the composition of the rumen microbiome. The microbial community in the rumen is influenced by dietary, anatomical, and physiological adaptations and has evolved alongside the diverse feeding strategies in different ruminant lineages (Hofmann, 1989; Langda et al., 2020). Peraza et al. (2024) also reported that diet has a significant effect on microbial diversity and abundance in beef cattle. In general, the eDNA from the rumen revealed that individual sheep variability and diet significantly influenced microbial composition and dynamics, as well as the test diet’s effect on *Methanobrevibacter*, which may lead to the need for further investigation.

### 4.3 Rumen microbial community diversity

The non-significant result (*p* = 0.65) in alpha diversity suggests that the treatments had comparable effects on microbial community richness and evenness. Peraza et al. (2024) found that alpha diversity indices showed no significant correlation with feed efficiency in beef cattle. Liu et al. (2021) reported that alpha diversity indices, including Sobs, Shannon, Simpson, Chao, and coverage, were not affected by feed supplement for grazing lactating yak. Langda et al. (2020) found that goats have greater bacterial alpha diversity than sheep, as shown by the Shannon index. A Shannon index value over 3 indicates high community diversity (Magurran, 2021). The higher Shannon index value, particularly in the Acacia and Ziziphus group, might be due to plant-origin feed rich in bioactive compounds that enhance the diversity of the rumen microbial population. Stoupi et al. (2010) and Wang et al. (2024) state that bioactive compounds influence gut microbiota indirectly by modulating immune responses. Some polyphenols affect immune cell function and cytokine production, altering the gut microbial environment.

Beta-diversity analysis measures the dissimilarity or distance between microbiome pairs (Knight et al., 2018). The beta diversity showed a gradual divergence in microbial communities over time. The significant effect at SD_90 aligns with observed shifts in microbial abundance (e.g., increased *Prevotella* in Control and BSG, sustained *Candidatus saccharimonas* in Acacia, transient peaks of *Methanosphaera* in Ziziphus). The rising R^2^ and significant *p*-value at SD_90 suggest that test feeds (Acacia, BSG, Ziziphus) caused greater differentiation in microbial communities compared to Control. Qu et al. (2020) and Shi et al. (2014) reported that the progression of microbial succession led to a remarkable increase in the diversity of the microbial community. Campbell et al. (2025) also reported that ruminal microbiota composition and diversity differed significantly over time.

Dietary influences can modify the rumen environment, promoting the growth of previously undetected or low-abundance genera. Different feed components, such as fibers, secondary metabolites, and proteins, are chosen to support specific microbial communities. Fibers are essential nutrients for maintaining gut microbiota diversity (Zhang, 2022). In a nutshell, the diversity indices indicate a growing influence of dietary treatments on microbiome composition as the *in vivo* trial progressed. The rising *R*^2^ also suggests that test feeds (Acacia, BSG, Ziziphus) caused greater differentiation in microbial communities compared to the Control, underscoring the role of feed in shaping rumen microbiomes.

### 4.4 Volatile fatty acids production

Acetate, propionate, and butyrate are the primary VFAs produced in the rumen, with acetate playing a crucial role in ruminant metabolism, which is dependent on key nutrients, other VFAs, amino acids, and glucose (Van Houtert, 1993). In the current study, the treatments influenced VFAs production, exhibiting distinct trends for each acid: acetate was highest in Acacia and lowest in BSG, propionate levels were higher in BSG, and butyrate levels were lower. Bhatt et al. (2022) found that including Acacia in sheep feed increased acetic acid concentration compared to the control diet, aligning with our study. Christodoulou et al. (2024) found that butyrate concentrations in BSG were lower while propionate levels remained similar in their study of four agro-industrial byproducts. In contrast, Miyazawa et al. (2007) observed no significant differences in the concentrations of acetic, propionic, and butyric acids among lactating dairy cows fed BSG compared to control diets. Dai et al. (2022) also reported that an *in vitro* assessment of mixed silage containing BSG showed a notable increase in lactic, acetic, and total volatile fatty acids. The varying relationship between treatment feed and VFAs might not be an appropriate measure for evaluating the progress or effects of treatments on ruminal fermentation (Hall et al., 2015).

Elevated propionate is believed to reduce CH_4_ production, while increased acetate leads to higher CH_4_ production. Danielsson et al. (2017) found that low CH_4_-emitting dairy cows exhibited a higher proportion of propionate in their VFA fermentation pattern, whereas high CH_4_-emitting cows showed a higher proportion of acetate and butyrate. In general, the treatment diets had a significant impact on VFA production in sheep, affecting the levels of acetate, propionate, and butyrate. The higher propionate in the BSG did not guarantee low CH_4_ intensity; instead, Ziziphus had low CH_4_ intensity. The VFA analysis revealed complex relationships with CH_4_ intensity that require further study with a larger sample size.

### 4.5 Correlation between rumen microbes, VFAs, and CH_4_ intensity

No notable correlations were found between CH_4_ intensity and specific Bacterial genera or VFAs. In contrast, the archaeal genus *Methanomicrobium* abundance strongly correlates with increased CH_4_ intensity, indicating its direct involvement in methanogenesis. Ding et al. (2012) reported a notably positive correlation between the methanogenic population and CH_4_ production. Danielsson et al. (2017) also found a positive correlation between dairy cows that emit low levels of CH_4_ and a higher abundance of the *Methanobrevibacter ruminantium* clade. However, Kittelmann et al. (2014) reported no correlation between high and low CH_4_ emitting sheep and the levels of methanogenic Archaea. Similarly, Shi et al. (2014) found that none of the microbial domains significantly correlated with sheep with low and high CH_4_ yields. Due to the significant variability in the relationship with total microbial abundance, the composition of the microbial community may hold more importance for CH_4_ emissions than merely its quantity (Tapio et al., 2017). Furthermore, rather than the abundance of genes, microbial gene expression plays a more crucial role in CH_4_ emissions from individual sheep (Shi et al., 2014).

These correlation findings highlight that CH_4_ emissions may be more closely linked to specific archaeal activity and microbial community composition rather than overall microbial abundance or fermentation end-products.

## 5 Conclusion

This study demonstrated that CH_4_ abatement feeds (Acacia, BSG, and Ziziphus) reduced the dominance of *Methanobrevibacter*, while increasing *Methanosphaera*, suggesting a shift in Archaeal dynamics. Bacterial genera, such as the *Rikenellaceae RC9* gut group and *Prevotella*, exhibited treatment-specific responses, although microbial diversity (as measured by the Shannon index) remained largely unaffected by diet. Time effects, however, significantly impacted beta-diversity and composition. VFA profiles reflected dietary impacts, with BSG enhancing propionate production while Acacia favored acetate. Despite these shifts, correlations between rumen microbes, VFAs, and CH_4_ intensity were generally weak, except for notable inter-VFA relationships and interactions between Archaeal genera. This implies that, although diet influences microbial communities and fermentation processes, the direct connections to CH_4_ reduction are complex and require further investigation. These findings enhance our understanding of how rumen microbes respond to feeding strategies and their potential role in reducing CH_4_ emissions in sustainable livestock production.

## Data Availability

The data presented in this study are publicly available. The data can be found here: https://doi.org/10.6084/m9.figshare.31018567.
