# Supplementary material for: Associations between volatile fatty acid profiles, methane emissions, and rumen microbiota in sheep fed Ethiopian forage
Source: Front Microbiol. 2026 Jan 22;16:1731623. doi: 10.3389/fmicb.2025.1731623 (PMC12874088; doi:10.3389/fmicb.2025.1731623)
Supplement: Supplementary file 1 [file Supplementary_file_1.pdf]

Supplementary Material

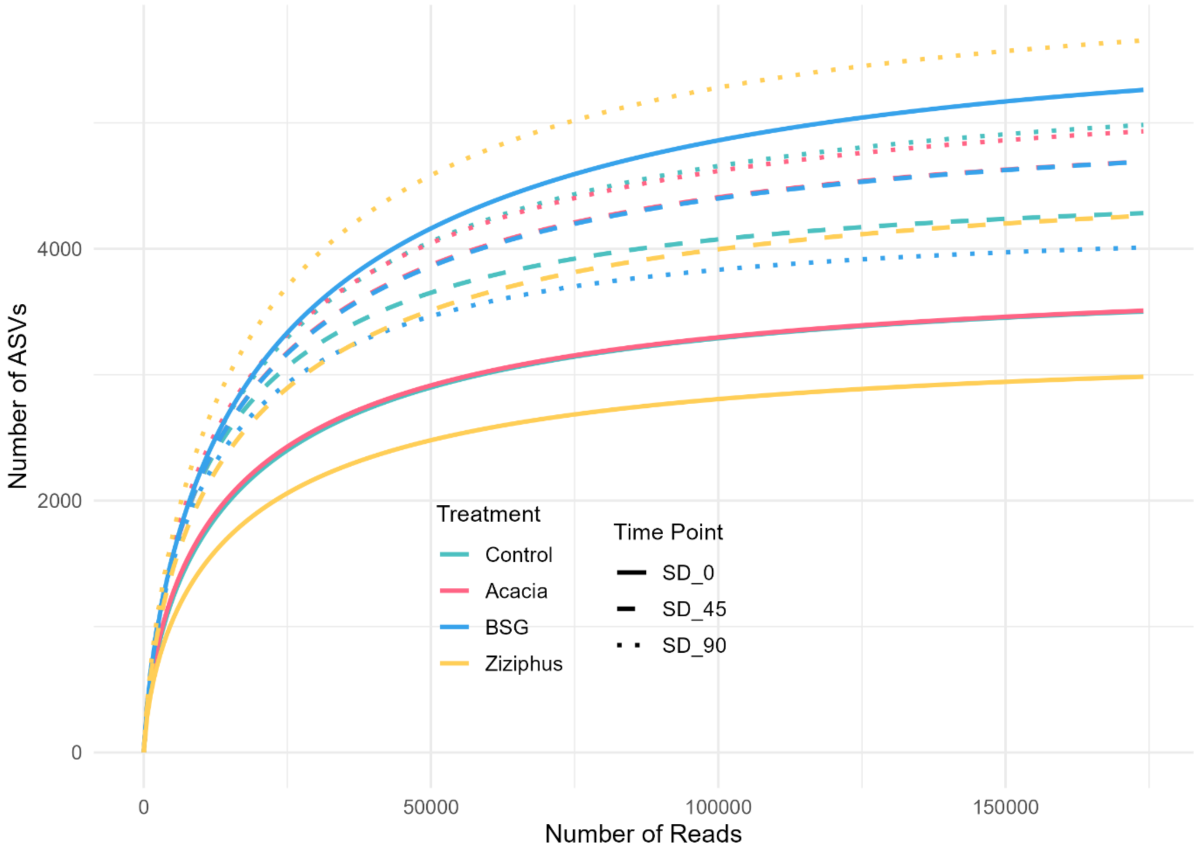

Figure S1. Rarefaction curves of bacteria showing the relationship between the number of reads per sample (sequencing depth) and the number of amplicon sequence variants (ASVs) across treatments and time points from eDNA of sheep rumen fluid. The four treatments are (Control, Acacia, BSG, and Ziziphus), and three time points (SD_0, SD_45, and SD_90). SD_0 represents samples collected before the treatment feed was introduced, while SD_45 and SD_90 correspond to samples collected on days 45 and 90 of the *in vivo* experiment following treatment initiation.

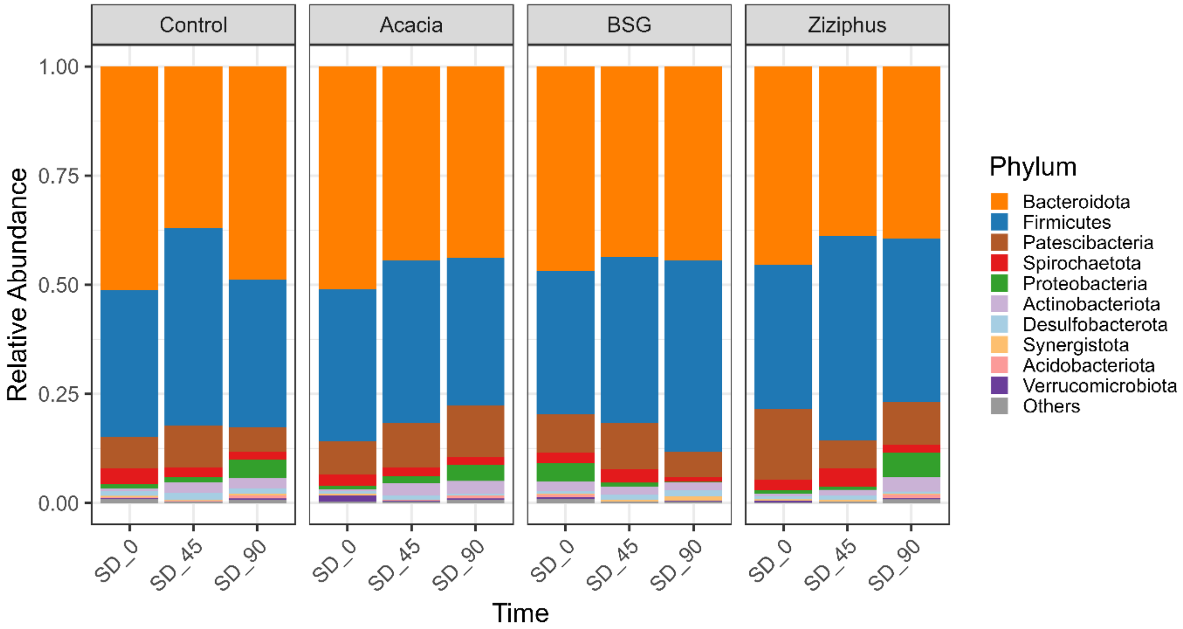

Figure S2. Stack plots representing the composition of bacterial phylum in eDNA of sheep rumen fluid after feeding of four diets (Control, Acacia, BSG, and Ziziphus) across three time points (SD_0, SD_45, and SD_90). SD_0 represents samples collected before the treatment feed was introduced, while SD_45 and SD_90 correspond to samples collected on days 45 and 90 of the *in vivo* experiment following treatment initiation. Others encompass the bacterial phyla below the top 10 phyla.

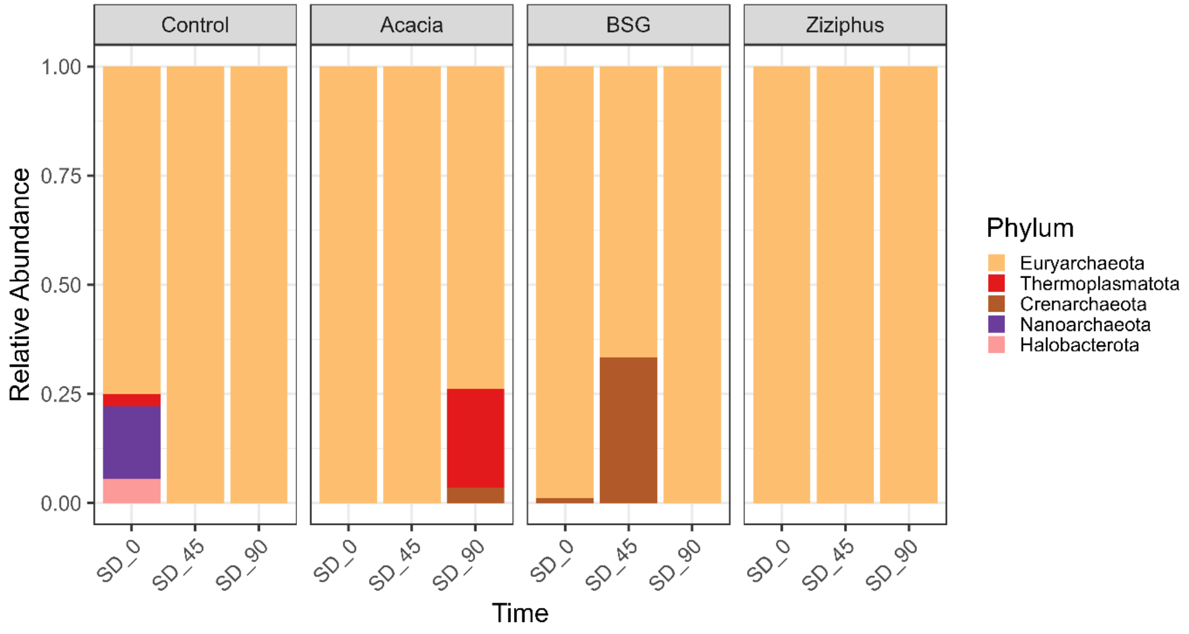

Figure S3: Stack plot of percentage share of the top archaeal phylum in eDNA of sheep rumen fluid after feeding of four diets (Control, Acacia, BSG, and Ziziphus) across three time points (SD_0, SD_45, and SD_90). SD_0 represents samples collected before the treatment feed was introduced, while SD_45 and SD_90 correspond to samples collected on days 45 and 90 of the *in vivo* experiment following treatment initiation.

Table S1. Relative abundance of the top ten genera within each domain (Bacteria and Archaea). For each domain, phylum-level relative abundances are shown, with the top ten genera listed alongside their proportion within the corresponding phylum.

BACTERIA			
Phylum	***% of phylum within domain***	***Genus***	***% of genus within phylum***
Bacteroidota	45.4	*Rikenellaceae RC9 gut group*	18.0
		Prevotella	17.0
		Prevotellaceae UCG-003	4.4
		Prevotellaceae UCG-001	3.2
Firmicutes	36.5	*Christensenellaceae R-7 group*	5.3
		Ruminococcus	2.1
		Succiniclasticum	1.9
		UCG-004	2.8
		NK4A214 group	3.1
Patescibacteria	9.1	*Candidatus saccharimonas*	8.9
Spirochaetota	2.4		
Proteobacteria	2.3		
Actinobacteriota	1.9		
Desulfobacterota	0.9		
Synergistota	0.3		
Acidobacteriota	0.3		
Verrucomicrobiota	0.3		
Others	0.6		
ARCHAEA			
Euryarchaeota	92.0	*Methanobrevibacter*	88.0
		Methanomicrobium	1.0
		Methanosphaera	11.0
Thermoplasmatota	3.0		
Crenarchaeota	3.0		
Nanoarchaeota	2.0		
Halobacterota	1.0		

1
